# Olive Mill Wastewater Valorization in Multifunctional Biopolymer Composites for Antibacterial Packaging Application

**DOI:** 10.3390/ijms20102376

**Published:** 2019-05-14

**Authors:** Laura Sisti, Grazia Totaro, Nicole Bozzi Cionci, Diana Di Gioia, Annamaria Celli, Vincent Verney, Fabrice Leroux

**Affiliations:** 1 Dipartimento di Ingegneria Civile, Chimica, Ambientale e dei Materiali, Università di Bologna, Via Terracini 28, 40131 Bologna, Italy; laura.sisti@unibo.it (L.S.); annamaria.celli@unibo.it (A.C.); 2 Department of Agricultural and Food Sciences, Università di Bologna, viale Fanin 42, 40127 Bologna, Italy; nicole.bozzicionci@unibo.it (N.B.C.); diana.digioia@unibo.it (D.D.G.); 3 Institut de Chimie de Clermont Ferrand (ICCF)—UMR 6296 Clermont-Auvergne Université, CNRS, 24 Avenue Blaise Pascal, 63177 Aubiere (CEDEX), France; vincent.verney@uca.fr (V.V.); fabrice.leroux@uca.fr (F.L.)

**Keywords:** multifunctional hybrid systems, olive mill wastewater, antibacterial properties, layered double hydroxides, bionanocomposites

## Abstract

Olive mill wastewater (OMW) is the aqueous waste derived from the production of virgin olive oil. OMW typically contains a wide range of phenol-type molecules, which are natural antioxidants and/or antibacterials. In order to exploit the bioactive molecules and simultaneously decrease the environmental impact of such a food waste stream, OMW has been intercalated into the host structure of ZnAl layered double hydroxide (LDH) and employed as an integrative filler for the preparation of poly(butylene succinate) (PBS) composites by in situ polymerization. From the view point of the polymer continuous phase as well as from the side of the hybrid filler, an investigation was performed in terms of molecular and morphological characteristics by gel permeation chromatography (GPC) and X-ray diffraction (XRD); also, the thermal and mechanical properties were evaluated by thermogravimetric analysis (TGA), differential scanning calorimetry (DSC), and dynamic thermomechanical analysis (DMTA). Antibacterial properties have been assessed against a Gram-positive and a Gram-negative bacterium, *Staphylococcus aureus* and *Escherichia coli*, respectively, as representatives of potential agents of foodborne illnesses.

## 1. Introduction

High-added-value compounds, such as pectin and polyphenols, can be recovered from agro-wastes and reused as food ingredients, cosmetics, or even in pharmaceutical preparation [1,2]. Many recent studies have been devoted to the valorization of agro-food by-products in order to address sustainable and environmental requirements [3,4,5]. However, the recovery of target components from waste implies the use of downstream and purification processes which are time consuming and costly as well as not environmentally friendly due to the use of huge amounts of water. An alternative approach consists of exploiting agro-wastes without any pretreatment, with the aim of preparing multifunctional materials, the development of which is indeed of great interest now for the industry, which is always looking for high-performance products obtainable through simple and low-cost processes. In the field of packaging, for example, where preserving the quality and increasing the safety of the products is of crucial importance, antibacterial, antioxidant, and/or anti-UV properties are greatly required. At the same time, in the packaging sector, biopolymers (from natural sources and/or biodegradable) are emerging on the market because of growing global pressure deriving from the extensively publicized effects of climate change, price increases of fossil materials, as well as depletion of global fossil resources [6]. However, bio-based materials do not achieve technical performance comparable to their fossil counterparts.

A straightforward strategy that allows for obtaining high-performance and multifunctional materials involves the use of organo-modified layered double hydroxides (LDHs) dispersed in polymeric matrices. LDHs are a class of anionic clays consisting of stacked brucite-type [Mg(OH)_2_] octahedral layers with water molecules and exchangeable anions in the interlayer region, offering a large variety of heterostructured materials [7]. Thanks to their great versatility as well as simple preparation, LDH host structures are often considered as a toolbox and their applications are strongly diversified, ranging from catalysis to biomedicine, cosmetics, functional additives, and/or stabilizers in polymer formulations, sorbents, and scavengers for pollutants [8,9].

Our research group is profoundly concerned with targeting green fillers in association with bio-based and biodegradable polymer. Our previous studies have demonstrated that the use of organo-modified LDHs may provoke a significant chain extension effect on polymer matrices with consequent improvement of their processability [10,11,12,13,14]. Moreover, suitable properties, such as antioxidant/antibacterial/anti-UV, due to the tethered/intercalated anions into the LDH, can be exploited, leading to multifunctional fillers. LDH acts as inorganic cargo hosting bioactive molecules and it protects them from thermal degradation, thus enhancing their thermal stability as well as allowing the preservation of their bioactivity, which is well-maintained under such a protective layer against injection/extrusion during polymer composite processing [15,16]. Therefore, multifunctional composite materials with antioxidant/antibacterial/anti-UV properties can be achieved.

In a previous work [17], olive mill wastewater (OMW), without any pretreatment, was successfully intercalated into a Zn_2_Al-LDH with the aim of enhancing the durability of polypropylene (PP) and poly(butylene succinate) (PBS) melt-blended composites. In the current study, the antibacterial and thermomechanical properties of similar systems have been further investigated. Therefore, the materials prepared can be suitable for antibacterial/antioxidant coating and packaging, as well as medical devices and household products.

OMW is a high-organic-load and recalcitrant waste stream of great environmental concern and its management is a very important issue in Mediterranean countries because of the huge amount of olives: more than 2.4 million tons/year, 90% of which is meant for olive oil production, thus generating up to 30 million m^3^ per year of OMW [17,18]. The composition of OMW consists of 80–90% water, 4–16% organic compounds (such as tyrosol, hydroxytyrosol, p-coumaric acid, ferulic acid, syringic acid, protocatechuic acid, vanillic acid tannins, anthocyanins, etc.), and 0.4–2.5% mineral salts (K, Ca, and Na). Due to their low partition coefficients, olive phenols are more soluble in water than in the oil phase. Thus, polyphenols are found in OMW at concentrations ranging from 0.03–11.5 g/L according to the processing system used for olive oil production [1].

Among bio-based polyesters, PBS is of particular interest. It is a biodegradable semicrystalline polymer, the pristine monomers of which (1,4-butanediol and succinic acid) can both be obtained from sugar fermentation. PBS is emerging in agriculture, consumer goods, and especially in the flexible packaging market [6]. Due to its thermal and mechanical characteristics, PBS ranks similar to PP for its strength (tensile strength of 34 MPa for PBS and 33 MPa for PP) and between low-density (LDPE) and high-density polyethylene (HDPE) for its stiffness (flexural modulus of 656 MPa for PBS, 176 MPa for LDPE, and 1070 MPa for HDPE) [19,20].

In detail, OMW was here used as an intercalating agent in layered Zn_2_Al-LDH (i.e., between its organo-modified platelets). Some phenol model molecules, as typical chemical structures of the main components present in OMW (namely, vanillic acid (VA), ferulic acid (FA), and protocatechuic acid (PA)), were also employed for comparison. Mixed systems were also prepared by simultaneous coprecipitation of two or three biomolecules to simulate a medium containing more than one bioactive compound, as in OMW. The organo-modified LDHs (5 wt%) were employed for the preparation of PBS composites through in situ polymerization. Both the organo-modified LDHs and the obtained composite samples were tested for their antimicrobial action against *Escherichia coli* and *Staphylococcus aureus*. Thermal and thermomechanical properties were investigated by thermogravimetric analysis (TGA), differential scanning calorimetry (DSC), and dynamic thermomechanical analysis (DMTA).

## 2. Results and Discussion

A full characterization of the prepared LDH samples, as well as the total organic compound (TOC) of the biowaste (4.51 ± 0.65 g GA eq/L, GA = gallic acid) were reported elsewhere [17]. Briefly, HPLC analysis revealed the presence of protocatechuic acid, vanillic acid, trans-cinnamic acid, gallic acid, and chlorogenic acid in OMW. FT-IR and X-ray analyses of the LDHs containing vanillic acid (Zn_2_Al/VA), ferulic acid (Zn_2_Al/FA), and protocatechuic acid (Zn_2_Al/PA) demonstrated the intercalation of the anions between the lamellae. Mixed systems were also prepared by simultaneous coprecipitation of FA and PA (Zn_2_Al/FA-PA) or VA, FA, and PA (Zn_2_Al/VA-FA-PA) to simulate a medium containing more than one bioactive molecule, as in OMW. Concerning such systems and the sample intercalated with OMW (Zn_2_Al/OMW), a selective interaction with some molecules was evident from FT-IR, but from X-ray diffraction analysis, it was concluded that the coprecipitation of LDH in the presence of OMW was not 100% efficient in yielding the LDH structure, giving rise to another inorganic material (γ-AlOOH) unable to trap organic guests. This was confirmed by a lower organic weight loss percentage from TGA in Zn_2_Al/OMW. Therefore, in such a sample (Zn_2_Al/OMW), the two structures (LDH and γ-AlOOH) were concomitantly present. The thermal protecting role of the LDH towards the anions was clear from TGA analysis because the thermal stability was enhanced after intercalation. This is a key point in this strategy because it allows the preservation of the bioactivity, which is well-maintained under such a protective layer during polymer composite processing.

**Scheme 1:**
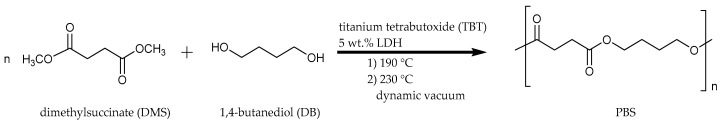
In situ polymerization of poly(butylene succinate) (PBS) bionanocomposites.

In the current study, 5 wt% of the organic/inorganic (O/I) hybrid fillers was employed to prepare PBS bionanocomposites by in situ polymerization, which is an effective method to reach a good state of dispersion for the LDH filler. Such loading was reported to be appropriate, in similar systems, to assure the persistence of bioactivity [16,17,21]. Scheme 1 shows the monomers employed and the conditions of the in situ polymerization process. The bionanocomposites have been labeled PBS:Zn_2_Al/X, according to the LDH employed.

The molecular weights of all composites, obtained by gel permeation chromatography (GPC) analysis, were high, into the range of 50 × 10^3^ < Mw < 90 × 10^3^ g/mol with polydispersity values coherent to common polyesters (M_w_/M_n_ ≈ 2) (Table 1).

PBS:FA/PA, without LDH, with 1 wt% of FA and 1 wt% of PA with respect to the polymer theoretical yield, was also prepared in order to better understand the beneficial role of LDH. Of course, during the polymerization, FA and PA competed with dimethyl succinate (DMS) in the condensation reaction with 1,4-butanediol (BD); therefore, a copolymer was formed. ^1^H NMR showed that only 0.32 mol% of both FA and PA had copolymerized. The results of GPC analysis indicated that oligomers were formed (M_w_ = 8 × 10^3^ g/mol) because FA and PA possess only one reactive functional group; therefore, they act as a chain stopper during macromolecular growth.

**Figure 1:**
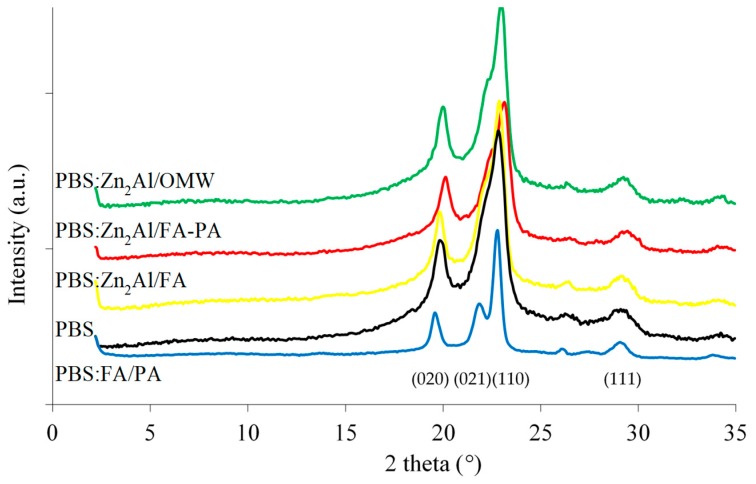
XRD profiles of PBS and some PBS:Zn_2_Al/X composites with indexing of the main reflections due to the crystalline structure of PBS.

The degree of dispersion of the organo-modified LDHs in the PBS matrix was evaluated by XRD analysis. In Figure 1, since the same results were obtained for all the samples, only some representative composite XRD patterns are reported, showing the diffraction lines characteristic of PBS and the absence of the diffraction peaks of the associated filler. This can be explained by a low crystallinity of the filler or a potential exfoliation occurring during thermal processing. All the characteristic peaks ascribable to the crystalline structure of PBS (020) (021) (110) (111) can be found in the 2θ range of 18°–30°, and no significant difference can be depicted with PBS free of filler. The copolymer presented narrow diffraction lines due to PBS crystallinity.

**Figure 2:**
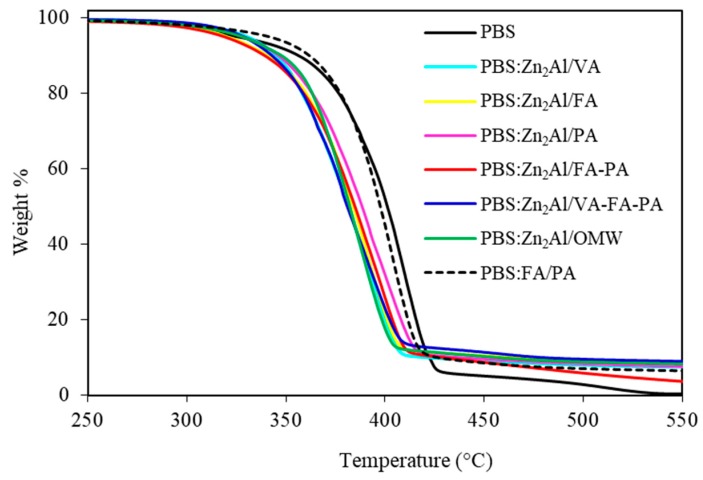
TGA profiles of PBS, PBS:FA/PA, and PBS:Zn_2_Al/X composites.

Table 1 and Table 2 list all the results from TGA and DSC analyses of the nanocomposites. From Figure 2, reporting the TGA thermograms of all the samples, a main decomposition process is depicted in the temperature range of 360–375 °C. Concerning the nanocomposites, the filler did not exert thermal stabilization upon the matrix because of the catalyzing effect of metals, as well as the polyester hydrolysis promoted by the water released during its thermal degradation. In any case, the thermal data reported (onset degradation (T_onset_), 10% mass loss (T^10^_D_), and maximum degradation temperatures (T_max_)) were consistently higher than the melting temperature of PBS (116 °C); therefore, such expected thermal degradation was overcome during processing. This is consistent with the literature [12,13]. As expected, the copolymer showed initial and maximum degradation temperatures close to PBS.

**Figure 3:**
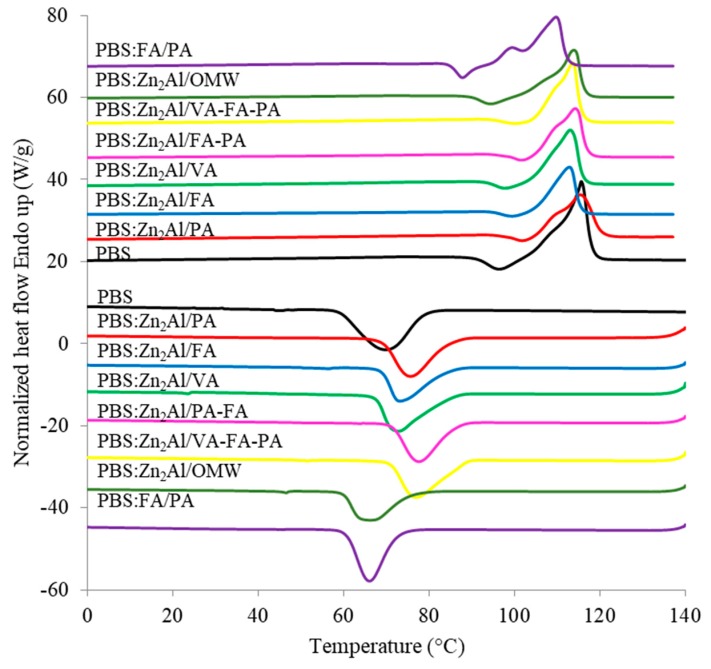
DSC profiles of PBS, PBS:FA/PA, and PBS:Zn_2_Al/X composites.

From the DSC analysis reported in Table 2, it can be noted that melting temperature (T_m_) values of the nanocomposites slightly decreased with respect to 116 °C (the melting temperature of PBS). Moreover, the peak shapes were complex and multiple endothermic peaks were present due to the melting–recrystallization process that generally occurs in polyesters (Figure 3) [22]. Concerning the cooling scan, most of the composites crystallized at higher temperatures with respect to PBS, while PBS:Zn_2_Al/OMW had a lower crystallization temperature (T_c_). However, all the composites presented lower enthalpy of crystallization (∆H_c_) with respect to PBS; therefore, the motion of the polymer chains in the melt were restricted by the platelets and the crystallization process was somehow hindered. Indirectly, this behavior confirmed a good degree of dispersion of the LDHs in the PBS matrix.

**Figure 4:**
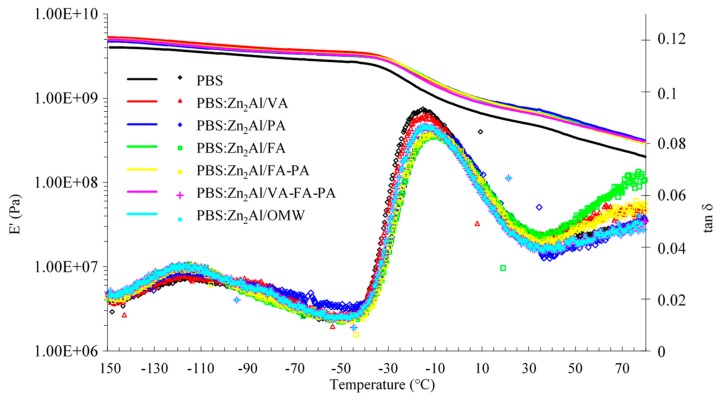
DMTA profiles of PBS and PBS:Zn_2_Al/X composites.

In order to evaluate the mechanical properties of the samples prepared, DMTA, which measures the response of a material to a cyclic deformation as a function of the temperature, was performed. Figure 4 shows the storage modulus E’ and tan δ, which is the ratio of the loss modulus to storage modulus, as a function of temperature. The nanocomposites, with respect to the homopolymer, presented superimposable curves, with E’ values slightly higher than PBS within the entire temperature range, which revealed the reinforcing role of the O/I hybrid filler upon PBS. Additionally, it highlighted an efficient interface interaction between the filler and the polymer, as well as a good state of dispersion of LDH platelets. The reinforcement effect of the fillers became more important above the glass transition temperature, when materials become soft, due to the restricted movement of the polymer chains [23]. At 20 °C, the fillers showed a medium E’ enhancement of 40%, and the maximum value was related to PBS:Zn_2_Al/PA (51%). With respect to DSC analysis, DMTA is a more sensitive technique to evaluate the glass transition temperature (T_g_) (Table 2). The bionanocomposites showed higher T_g_ values with respect to PBS (−16.3 °C), which was due to the restriction of the movements of the macromolecular chains because of their interaction with the nanosheets of organo-modified LDH fillers.

**Figure 5:**
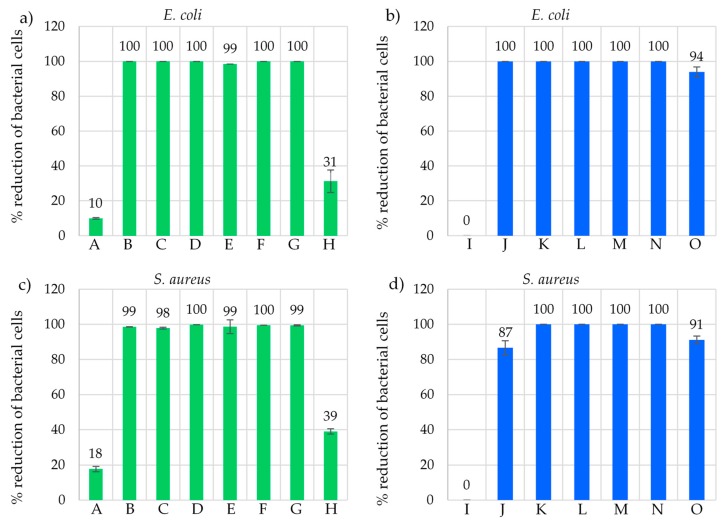
Antibacterial properties of PBS, PBS:FA/PA, bionanocomposites (left side), and layered double hydroxides (LDHs) (right side) against *Escherichia coli* (**a**,**b**) and *Staphylococcus aureus* (**c**,**d**). A = PBS, B = PBS:Zn_2_Al/VA, C = PBS:Zn_2_Al/FA, D = PBS:Zn_2_Al/PA, E = PBS:Zn_2_Al/FA-PA, F = PBS:Zn_2_Al/VA-FA-PA, G = PBS:Zn_2_Al/OMW, H = PBS:FA/PA, I = PuralMg61, J = Zn_2_Al/VA, K = Zn_2_Al/FA, L = Zn_2_Al/PA, M = Zn_2_Al/FA-PA, N = Zn_2_Al/VA-FA-PA, and O = Zn_2_Al/OMW.

The antibacterial properties of both O/I hybrid fillers and nanocomposites were checked against a Gram-positive bacterium, *S. aureus*, and a Gram-negative one, *E. coli*, which are potential agents of foodborne illnesses causing serious infections [15] and ubiquitous widespread bacteria [24,25] (Figure 5). The activity was calculated using Equation (1), expressed as a percentage of the reduction of viable cells in the assayed sample compared with a negative control consisting of a bacterial culture grown in the absence of any material. As a prerequisite for the whole set of experiments, reference materials (PuralMg61 and PBS) were assayed for the antimicrobial evaluation tests, showing no antibacterial effectiveness for PuralMg61 and an antimicrobial activity of 10% and 18% for PBS in the presence of *E. coli* and *S. aureus*, respectively. Hybrid LDH powders were characterized by a strong antibacterial activity (cell mortality rate = 100%) against *S. aureus* and *E. coli*. Slightly lower values were recorded for Zn_2_Al/VA versus *S. aureus* (87%) and Zn_2_Al/OMW (94% and 91% versus *E. coli* and *S. aureus*, respectively). In the latter case, the presence in the waste of different phenolic compounds, as well as of other substances, might have somehow interfered with the antibacterial activity, causing a slightly lower mortality with respect to the use of the pure compounds. However, the percentage of mortality was also high (>90%) with the OMW-containing samples. These results are in agreement with literature data [26,27,28] and confirm the strong antimicrobial activity associated with OMW phenolic compounds.

Considering the PBS nanocomposites, the results obtained using 50 mg of each sample on 500 μL of bacterial cells (10^5^ colony forming units (CFU)/mL) (Figure 5a,c) highlight that the antimicrobial activity was maintained in the final products, reaching values of 100% of mortality against *E. coli* and close to 100% against *S. aureus*. In addition, two amounts of PBS:Zn_2_Al/OMW (50 and 10 mg) were tested using the same suspension of *E. coli* cells (500 μL), reaching in both cases a cell mortality rate of 100%. The slight differences in the antibacterial properties could be due to the different architectures of the outer layers of Gram-positive and -negative bacteria that may favor the antibacterial action of lipophilic molecules in Gram-negative cells. Indeed, *E. coli* has an extra lipopolysaccharide membrane outside a thin cell wall layer, while a thick peptidoglycan wall represents the external envelope in Gram-positive bacteria [15]. Phenols are reported both to destroy the external Gram-negative layers and to pass through this layer, thus exerting their biocidal activity in the cytoplasm [29]. Indeed, they act specifically on the cell membrane and inactivate intracytoplasm enzymes by forming unstable complexes.

On the other hand, as expected, the copolymer PBS:FA/PA did not present any significant antibacterial activity, thus highlighting the role of LDH in protecting the bioactivity of the organic modifiers during the polymerization process. Indeed, the inorganic host thermally protected the intercalated molecules, thus preserving their bioactivity.

**Figure 6:**
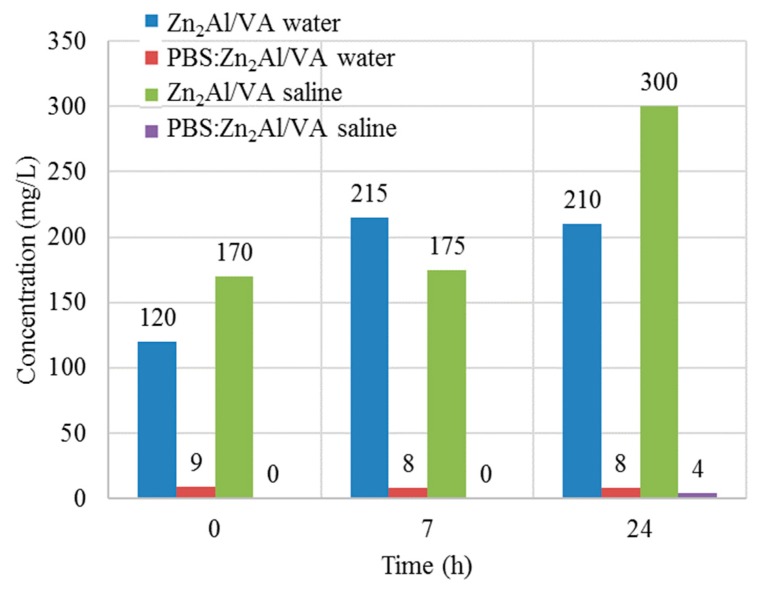
Concentration of vanillic acid evaluated by HPLC analyses in water or saline in which samples have been immersed.

To evaluate if such high activity in the case of the hybrid LDH materials could have been due to phenolic acids potentially released from the LDH surface into the test medium, release studies of VA (as a target bioactive molecule) from Zn_2_Al/VA and PBS:Zn_2_Al/VA were carried out (Figure 6). The results show a maximum release of 300 mg/L from Zn_2_Al/VA in saline but, most importantly, a negligible release was obtained for the corresponding nanocomposite (maximum 9 mg/L). The released VA could not be responsible for the inhibitory activity against the target microorganisms, considering that the minimal inhibitory concentration (MIC) of VA is 500 mg/L versus *E. coli* (although a partial inhibition can also be seen at 300 mg/L) and higher than 500 mg/L versus *S. aureus* (i.e., higher amounts with respect to those released from the specimens (Table 3)).

The potential of using OMW as an antimicrobial agent was confirmed by the data obtained with LDH and PBS nanocomposites containing the waste as it is. These results demonstrated that it is possible to obtain optimal antimicrobial performances also by using OMW without any pretreatment, potentially leading to a true recycling and valorization of this biowaste in the development of multifunctional materials.

## 3. Materials and Methods

### 3.1. Materials

BD, DMS, sodium hydroxide, aluminum nitrate Al(NO_3_)_3_∙9H_2_O, zinc nitrate Zn(NO_3_)_2_∙6H_2_O, VA, FA, PA, ethanol, and titanium tetrabutoxide (TBT) were purchased from Aldrich Chemical (Darmstadt, Germany). All the materials were used as received. Nutrient broth and plate count agar were from Oxoid, Basingstoke, UK. McFarland turbidity standards were from Biolife, Milano, Italy. The OMW was furnished by Sant’Agata d’Oneglia (Imperia, Italia) and concentrated prior to use: from 10 L, 1 L was obtained. Its main chemical features were: chemical oxygen demand (COD) of 43.5 ± 1.6 g/L; TOC of 4.51 ± 0.65 g GA eq/L; and total suspended solids (TSS) of 39,200 ± 4,808 mg/L.

### 3.2. LDH Synthesis

The LDHs were prepared by coprecipitation following a procedure described elsewhere [17] with OMW, VA, FA, and PA. Two co-intercalated LDHs with mixtures of FA + PA and VA + FA + PA were also prepared. Briefly, 50 mL of deionized water solution containing 31.2 mmol of Zn(NO_3_)_2_∙6H_2_O and 15.6 mmol of Al(NO_3_)_3_∙9H_2_O was added dropwise for 3 h in a reactor containing 62.4 mmol of the anion in 100 mL of ethanol/deionized water (60/40) under vigorous stirring. In the case of OMW, 60 mL was used. The pH was maintained at 9.5 (±0.1) through the addition of NaOH solution. The reaction was carried out under nitrogen atmosphere and stirred for 3 h at room temperature. The solid material was separated and submitted to three cycles of deionized washing/ethanol centrifugation. The samples were labeled as Zn_2_Al/X, where X is the anion. A full characterization of the LDHs (X-ray diffraction, FT-IR, and TGA analysis) is reported elsewhere [17].

### 3.3. Bionanocomposite Preparation

Bionanocomposites with 5 wt% loading of the organo-modified LDHs were prepared by in situ polymerization following a common procedure already reported [30]. As an example, the procedure for preparing PBS:Zn_2_Al/VA was as follows: a round-bottomed, wide-neck glass reactor (250-mL capacity) was loaded with BD (29.6 g, 328 mmol), TBT (0.0585 g, 0.172 mmol), and 2.35 g of Zn_2_Al/VA (previously dried overnight at 105 °C), corresponding to 5 wt% with respect to the polymer theoretical yield. The reactor was closed with a three-necked flat flange lid equipped with a mechanical stirrer and a torque meter which gave an indication of the viscosity of the reaction melt. The system was then connected to a water-cooled condenser and immersed in a thermostatic salt-bath at 190 °C under vigorous stirring. After 1 h, DMS (40.0 g, 274 mmol) was added and the mixture was kept at 190 °C until all the methanol distilled off. Then, the reactor was connected to a liquid-nitrogen-cooled condenser and a dynamic vacuum was applied down to 0.5 mbar while the temperature was increased up to 230 °C. When the torque of the melt was around 7–8 mN, a highly viscous, light-brown, and transparent melt was discharged from the reactor. For comparison, a PBS homopolymer was also synthesized, as well as PBS:FA/PA, without LDH with 1 wt% of FA + 1 wt% of PA. The molecular structure of PBS was confirmed by ^1^H NMR. ^1^H NMR on PBS:FA/PA was conducted on a purified sample.

### 3.4. Antibacterial Properties

The antimicrobial properties were assessed by evaluating the survival of bacterial cells exposed to the prepared samples: LDHs intercalated with phenolic acids or OMW, in order to check the antibacterial activity of the active principles, and PBS with 5 wt% LDHs, to test the efficacy of the final bionanocomposites. The two microorganisms used were *E. coli* ATCC 8739 and *S. aureus* ATCC 6538. Bacteria were grown aerobically in nutrient broth for 16 h at 37 °C. The bacterial culture obtained was centrifuged at 7000 rpm for 10 min, washed in sterile saline (0.9% *w*/*v* NaCl), and resuspended in the same solution in order to obtain ≈10^5^ CFU/mL.

The experiments on both LDHs and composites were performed using 50 mg of powder of each sample, which were put in contact with 500 µL of cells (≈10^5^ CFU/mL in saline) at room temperature (about 23 ± 1 °C) for 24 h. The same experiment was preliminarily done with a commercial LDH sample (PuralMg61) and a commercial PBS (i.e., with no added compounds). The composites were sieved in order to achieve a homogeneous, fine particle size. After the incubation, each sample was serially diluted (1:10) and the dilutions were plated on plate count agar. After incubation of the plates at 37 °C for 24 h, the number of colonies corresponding to the number of viable cells was determined, after averaging using triplicates, through a modification of the equation reported by Lala et al. [31]:

R % = [(B-A)/B] × 100(1)

where R % is the percentage of reduction of viable cells, A is the average number of viable cells obtained after 24 h of contact with sample powders, and B is the average number of viable cells after 24 h of incubation of a bacterial cell suspension in the absence of any material. As already mentioned, the commercial powders with no added compounds were also tested for their antimicrobial activity against the same strains.

An additional experiment was carried out only on the bionanocomposite intercalated with OMW (PBS:Zn_2_Al/OMW) in order to evaluate the antimicrobial activity with a less concentrated amount of sample: 10 mg of powder was put in contact with 500 μL of *E. coli* cells (≈10^5^ CFU/mL in saline) and incubated like the previous experiment.

#### 3.4.1. MIC of Vanillic Acid as a Target Compound

Different concentrations of vanillic acid were tested in order to evaluate the minimal concentration that inhibits the growth of *E. coli* and *S. aureus*. A stock solution of the assayed compound was prepared at a concentration of 10,000 mg/L in ethanol 70% (*v*/*v* of water). The concentrations tested were: 500, 300, 150, and 0 mg/L. Bacterial growth in the presence of vanillic acid at different concentrations was monitored by evaluating the turbidity of the culture after 24 h of incubation at 37 °C using a densitometer (DEN1-Biosan, Riga, Latvia), which evaluated turbidity using the MacFarland scale. Commercial McFarland standards (0.0–0.6 McFarland units) were used for calibrating the densitometer. The sample for MIC testing consists of 3 mL of nutrient broth, to which 100 µL of a culture grown overnight (10^8^ CFU/mL) and the appropriate volume of vanillic acid from the stock solution were added. The assay was performed in duplicate.

#### 3.4.2. Release Study

Release studies of vanillic acid from LDH and nanocomposite to the surrounding environment were conducted in water and saline on samples containing Zn_2_Al/VA and the corresponding composite (50 mg/500 μL) under stirring for 24 h at room temperature. Small aliquots of the supernatants were analyzed by HPLC at 0, 8, and 24 h of incubation.

### 3.5. Measurements

^1^H NMR spectra were recorded on a Varian Mercury 400 spectrometer (chemical shifts are in part per million downfield from tetramethylsilane; the solvent used was CDCl_3_.

GPC measurements were performed on a HP 1100 Series using a PL gel 5-µm Minimixed-C column with chloroform as the eluent and solvent for polymer samples. A refractive index detector was used and a calibration plot was constructed with polystyrene standards.

TGA was performed in air atmosphere using a TGA7 apparatus (gas flow of 30 mL/min, Perkin Elmer, Waltham, Massachusetts, USA) at a 10 °C min^−1^ heating rate from 50 to 900 °C for all the samples. The T_onset_, T^10^_D_, and T_max_ were measured.

DSC was carried out, under nitrogen flow, using a DSC6 (Perkin Elmer, Waltham, Massachusetts, MA, USA). To erase any previous thermal history, the samples (ca. 10 mg) were first heated at 20 °C min^−1^ to 140 °C, kept at a high temperature for 2 min, cooled down to −60 °C at 10 °C min^−1^, and heated from −60 to 150 °C at 10 °C min^−1^ (second scan). During the cooling scan, the T_c_ and ΔH_c_ were measured. During the second heating scan, the T_g_, T_m_, and corresponding enthalpy (ΔH_m_) were measured.

Nanocomposite powder samples were analyzed by XRD in steps of 0.07° over a 2θ range of 2.1°–35° at room temperature with a Bragg/Brentano diffractometer (XPERT-PRO, PANalytical, Royston, United Kingdom) with Cu Kα radiation (λ = 0.154 nm, monochromatization by primary graphite crystal) generated at 40 mA and 40 kV.

Physical and mechanical properties were determined using a DMTA IV Dynamic Mechanic Thermo analysis instrument (Rheometric Scientific, Reichelsheim Germany) with a dual cantilever testing geometry. Typical test samples were bars (33 mm × 8 mm × 2 mm) obtained by injection molding at 140 °C using a Minimix Molder. The analysis was carried out from −150 to 80 °C (heating rate of 3 °C min^−1^, frequency of 3 Hz, and strain of 0.01%).

## 4. Conclusions

Layered double hydroxides intercalated by vanillic acid, ferulic acid, protocatechuic acid, and olive mill wastewater were dispersed in PBS through in situ polymerization. The thermal protecting role of the inorganic host towards the bioactive molecules was here confirmed. DMTA demonstrated that all the fillers endowed PBS with a pronounced reinforcing effect. In particular, the nanocomposite with Zn_2_Al/PA at 20 °C displayed an elastic modulus E’ enhanced by more than 50% compared with PBS free of filler.

All the nanocomposites presented impressive antibacterial activity as well as negligible release of the active agent under the tested conditions. Therefore, these preliminary results highlight the possibility of using OMW as a potential agent to confer antimicrobial activity to nanocomposites, with an important additional value of decreasing the environmental impact of an offensive food stream. In addition, besides the pronounced antimicrobial properties, the phenolic compounds in OMW have proved to exert beneficial effects on human health: PA is reported to be an anticancer agent [32] as well as FA, which has anti-inflammatory, antiviral, and immunoprotective properties. Both molecules can inhibit cancer by scavenging reactive oxygen species or being involved in the cell cycle upon cellular uptake [33]. These points further support the use of OMW phenolic compounds for applications addressed to human health and care, taking also into account their previously demonstrated antioxidant properties [17].

Reminiscent of the use of other biowastes, such as lignosulfonated LDH blends with plasticized starch and thermoplastics [34], this could represent a general strategy that is potentially employable with other biowastes.

## Figures and Tables

**Table 1:** Gel permeation chromatography (GPC) and thermogravimetric analysis (TGA) results of PBS, PBS:ferulic acid (FA)/protocatechuic acid (PA) and PBS:Zn_2_Al/X composites.

Sample	M_w_ (× 10^−3^ g mol^−1^) ^a^	M_w_/M_n_^a^	T_onset_ (°C) ^b^	T^10^_D_ (°C) ^b^	T_max_ (°C) ^b^
PBS	69	2.4	380	363	406
PBS:Zn_2_Al/VA	91	2.4	353	344	393
PBS:Zn_2_Al/FA	47	2.2	357	340	393
PBS:Zn_2_Al/PA	49	2.3	355	347	389
PBS:Zn_2_Al/FA-PA	61	2.3	355	338	395
PBS:Zn_2_Al/VA-FA-PA	68	2.5	345	341	387
PBS:Zn_2_Al/OMW	61	2.4	355	347	387
PBS:FA/PA	8	2.7	376	360	403

^a^ Determined by GPC in CHCl_3_; ^b^ Determined by TGA under air flow.

**Table 2:** Thermomechanical results of PBS, PBS:FA/PA, and PBS:Zn_2_Al/X.

Sample	T_g_ (°C) ^a^	T_g_ (°C) ^b^	T_c_ (°C) ^c^	∆H_c_ (J·g^−1^) ^c^	T_m_ (°C) ^b^	∆H_m_ (J·g^−1^) ^b^
PBS	−16.3	−33	70	72	116	77
PBS:Zn_2_Al/VA	−14.4	−35	73	64	113	64
PBS:Zn_2_Al/FA	−8.9	−36	73	52	113	47
PBS:Zn_2_Al/PA	−8.9	−34	76	53	115	48
PBS:Zn_2_Al/FA-PA	−8.9	−36	77	55	114	51
PBS:Zn_2_Al/VA-FA-PA	−13.4	−36	77	61	114	58
PBS:Zn_2_Al/OMW	−13.4	−36	66	51	114	57
PBS:FA/PA	/	−30	66	62	110	67

^a^ Determined by dynamic thermomechanical analysis (DMTA); ^b^ Determined by differential scanning calorimetry (DSC) during the second heating scan; ^c^ Determined by DSC during the cooling scan from the melt at 10 °C min^−1^.

**Table 3:** Minimal inhibitory concentration (MIC) of vanillic acid (taken as a target bioactive molecule) vs. *E. coli* and *S. aureus*. Strain growth is reported as turbidity in the McFarland scale. Values represent the difference between the turbidity measured after 24 h of incubation at 37 °C and that detected at the beginning of the incubation.

Vanillic Acid (mg/L)	Growth after 24 h of Incubation	
	*E. coli*	*S. aureus*
500	0.175 ± 0.025	1.385 ± 0.016
300	0.495 ± 0.015	3.235 ± 0.031
150	1.240 ± 0.023	3.585 ± 0.021
75	2.800 ± 0.018	4.24 ± 0.017
0	3.250 ± 0.026	4.935 ± 0.024
